# Association analysis of obesity and hypertension and research on ethnic heterogeneity: a cross-sectional study based on different populations in Northwest China

**DOI:** 10.1186/s12889-025-25971-4

**Published:** 2025-12-15

**Authors:** Zhaohang Li, Jingjing Wang, Yujie Yuan, Rui Liu, Caixia Xi, Yonglan Li

**Affiliations:** 1 College of Life Science and Technology, Inner Mongolia Normal University, Hohhot, China; 2 Key Laboratory of Biodiversity Conservation and Sustainable Utilization in Mongolian Plateau, College and University of Inner Mongolia Autonomous Region, Hohhot, China

**Keywords:** Ethnic heterogeneity, Obesity, Hypertension, Health disparities, Health equity

## Abstract

**Background:**

Hypertension is a major global health issue with increasing prevalence, particularly in China. It is closely linked to obesity, which is a key risk factor for hypertension through mechanisms such as insulin resistance and inflammation. However, research on the relationship between obesity and hypertension in Northwestern China, particularly among diverse ethnic populations, remains limited.

**Methods:**

A cross-sectional study involving 6,186 adults from 10 ethnic groups was conducted between 2018 and 2023 in Gansu, Qinghai, and Inner Mongolia. Anthropometric data, including BMI, WC, BFP, and VFL, were collected. Hypertension was diagnosed on the basis of blood pressure measurements or prior diagnosis. Poisson regression and logistic regression models were used to analyse associations between obesity and hypertension, accounting for ethnic and demographic factors.

**Results:**

Statistically significant differences in obesity-related indicators were observed among ethnic groups. The composite obesity group presented the highest hypertension prevalence (65.5%), followed by the central obesity group (60.8%), generalized obesity group (43.2%), and non-obese group (35.5%). All obesity-related indicators (BMI, WC, VFL, and BFP) were positively associated with the odds of hypertension, with BFP demonstrating the strongest effect. RCS analysis revealed a linear association of WC and BFP with hypertension and a nonlinear association of BMI and VFL with hypertension.

**Conclusions:**

In conclusion, our study demonstrates that composite obesity is a more effective indicator for hypertension than other phenotypes. The significant ethnic variations observed underscore the necessity of incorporating ethnic heterogeneity into future prevention strategies and screening protocols, utilizing indicators such as BMI, WC, VFL, and BFP.

## Introduction

Hypertension is a major chronic disease that poses a significant threat to public health globally [1]. It is characterized by sustained elevated arterial blood pressure, which leads to chronic damage to the cardiovascular system [2]. According to the World Health Organization (WHO), approximately 1.28 billion adults worldwide suffer from hypertension, with nearly half of these individuals remaining undiagnosed or untreated [3]. In China, the burden of hypertension is particularly pronounced, with a staggering 144.4% increase in hypertension prevalence among youth aged 20–39 years between 1991 and 2015 [4], breaking the longstanding belief that hypertension is a condition exclusive to elderly individuals. Hypertension is not only a primary risk factor for cardiovascular diseases but also closely linked to other severe health conditions, such as stroke [2], renal failure [5], and retinopathy [6], severely impacting both the quality of life and life expectancy of affected individuals.

Obesity is one of the key risk factors for hypertension [7], with the two conditions interacting through mechanisms such as insulin resistance [8], inflammatory responses [9], and activation of the renin-angiotensin system [10]. The World Obesity Federation (WOF) projects that by 2035, the global population of individuals affected by obesity will reach 1.9 billion, accounting for 25% of the global population, with a notable increase in childhood obesity rates [11]. The global obesity epidemic and its associated health risks present a growing challenge to healthcare systems and impose a heavy financial burden on society [12, 13]. Common anthropometric measurements, such as body mass index (BMI) and waist circumference (WC), are widely used as indicators of general [14] and abdominal obesity [15], respectively. However, over time, one limitation of BMI is its inability to distinguish between different somatotypes and body compositions [16], a limitation also applicable to waist circumference. Research has shown that individuals with a normal BMI but a high body fat percentage face increased cardiovascular metabolic risk [17]. Waist circumference is a crucial indicator of visceral fat distribution [18] and plays a decisive role in the development of atherosclerosis [19]. In contrast, body fat percentage (BFP) quantifies the overall proportion of body fat, and the visceral fat level (VFL) provides a precise measure of visceral fat accumulation. Joint analyses of BFP with BMI and VFL with WC respectively may more comprehensively reveal the associations between obesity phenotypes and hypertension.

Previous studies have partially explored the relationships between obesity-related indicators and hypertension in other regions [20, 21], but few such studies have focused on ethnic populations in northwestern China. Given the unique genetic background, dietary habits, and lifestyle of Northwestern ethnic groups, their obesity phenotypes and metabolic characteristics may differ from those of other populations. Therefore, this study aims to analyse the associations between different obesity phenotypes and hypertension in Northwestern China and to explore whether obesity-related indicators can serve as risk markers for hypertension, with the goal of filling the research gap in health associations in Northwestern China.

## Methods

### Participants

This study conducted a cross-sectional survey between 2018 and 2023 in the regions of Gansu, Qinghai, and Inner Mongolia, involving a total of 6,186 adults (2,773 males, 3,413 females) from 10 different ethnic groups. A total of 7 anthropometric indicators were collected from the participants. This study received approval from the Ethics Committee of the supporting institution, and the ethical approval form is provided in the appendix. All participants signed informed consent forms, and the study adheres to the ethical principles outlined in the *Declaration of Helsinki*. The inclusion criteria were being a native resident for three generations or more and having no physical disabilities. The age range of the participants was 18–90 years, with an average age of 51.2 ± 15.6 years for males and 49.5 ± 14.0 years for females.

### Measurements

The participants were measured barefoot and in light clothing. Trained members of the research team used an anthropometric tape to measure waist circumference twice at the midpoint of the line connecting the lower ribs and the iliac crest, and the average was recorded. Height was measured via a Martin anthropometer (FanYing HXBM-19, Jiangsu, China). Subsequently, body composition was assessed via bioelectrical impedance analysis with a segmental body composition monitor (TANITA BC-601, Japan). Body mass index, body fat percentage, and visceral fat level were calculated on the basis of the participants’ age, height, and sex. The monitor simultaneously classified body fat percentage and visceral fat level as low, normal, or high (classification criteria: 1 = low; 2 = normal; ≥3 = high). After resting in a seated position for at least 10 min, each participant’s systolic blood pressure (SBP) and diastolic blood pressure (DBP) were measured on the right arm via an automatic blood pressure monitor (OMRON HEM-7071, Shenyang, China). All the measurements were conducted in strict accordance with the *Anthropometric Methods* [22] and the *Anthropometry Manua*l [23].

### Criteria for diagnosing obesity and hypertension and obesity phenotyping

General obesity tendency was defined according to the WHO Asia-Pacific BMI classification, with a BMI ≥ 25 kg/m² considered obese [24]. Central obesity tendency was assessed via the waist circumference thresholds established by the International Diabetes Federation (IDF) for the Asian population [25]: waist circumference ≥ 0.90 m for males and ≥ 0.80 m for females. Based on integrated diagnostic criteria that combine both anthropometric measurements and body composition indicators, this study defined four mutually exclusive obesity phenotypes. The central obesity group was defined as males with a waist circumference ≥ 0.90 m or females with a waist circumference ≥ 0.80 m, along with a visceral fat level classification of ≥ 3. The generalized obesity group was defined as having a BMI ≥ 25 kg/m² and a body fat percentage classification of ≥ 3. The composite obesity group was characterized by meeting both the criteria for central and generalized obesity, whereas the non-obese group comprised participants who met neither the criteria for central obesity nor those for general obesity.

Participants were required to refrain from smoking and caffeine intake for at least 30 min prior to blood pressure measurement. Hypertension was determined on the basis of the following criteria [25]: (1) a prior diagnosis of hypertension or ongoing antihypertensive treatment or (2) SBP ≥ 130 mmHg or DBP ≥ 85 mmHg.

### Statistical analysis

The normality of the data distribution was assessed via the Kolmogorov-Smirnov test. Sex differences in obesity-related indicators were tested via the Mann-Whitney U test, whereas intergroup differences were assessed via analysis of variance (ANOVA) or the Kruskal-Wallis test for normally or nonnormally distributed continuous variables, respectively. Categorical variables were compared via the chi-square test. All the statistical analyses were performed via SPSS (version 18.0; Armonk, New York, USA), with the significance level set at 5%.

A Poisson regression with robust variance was employed via Stata software (version 18.0; Stata Corp, College Station, TX, USA) to examine the associations between different obesity phenotypes and hypertension. A robust variance estimator was employed to correct for potential over-dispersion, so that accurate standard errors and confidence intervals could be obtained [26]. The participants were stratified by sex, age, and ethnicity, and interaction tests were conducted to assess the impact of these factors on the associations.

To handle missing data for covariates, we employed multiple imputation via chained equations to increase the statistical power and reduce selection bias [27]. Violin plots were used to visualize the blood pressure distribution across different obesity phenotypes. Obesity-related indicators (WC, BMI, VFL, and BFP) were divided into four quartiles (Q1-Q4) respectively, with Q1 serving as the reference group. A logistic regression model in R (version 4.4.3; R Core Team, Vienna, Austria) was used to evaluate the associations between obesity-related indicators and hypertension. Multicollinearity diagnostics showed all covariate variance inflation factors (VIFs) were below 4.00. For the obesity-related indicators (BMI: 4.80; WC: 3.42; VFL: 2.75; BFP: 2.32), all VIFs were below the threshold of 5.00, indicating that multicollinearity does not substantially bias the regression estimates [28]. A restricted cubic spline (RCS) was used to analyse the dose‒response relationship between obesity-related indicators and hypertension. To address multiple testing across the four obesity-related indicators, we applied the Benjamini-Hochberg false discovery rate (FDR) correction separately to *P* values from univariate and multivariate analyses.

## Results

### Analysis of differences in obesity-related indicators among ten ethnic groups in the Northwestern China

The results revealed statistically significant differences (*P* < 0.01, Table 1) in obesity-related indicators among the 10 ethnic groups in Northwest China. The Yugur ethnic group exhibited relatively high levels of BMI, VFL, and BFP, while the Salar ethnic group showed a high WC value. In contrast, the Dongxiang and Bonan ethnic groups had relatively low values across most obesity indicators. Additionally, WC and BFP demonstrated the greatest variation among the different ethnic groups. With the exception of WC in the Tu and Bonan ethnic groups, both waist circumference and visceral fat level were significantly higher in males than in females, whereas body fat percentage was significantly lower in males.

**Table 1:** Obesity-related indicators among ethnic populations in Northwest China

	Male				Female			
	BMI	WC	VFL	BFP	BMI	WC	VFL	BFP
Yugur	26.8 ± 3.9	0.91 ± 0.09	14.9 ± 3.7	26.0 ± 5.6	27.6 ± 4.4	0.88 ± 0.10^**^	8.9 ± 2.7^**^	40.1 ± 6.8^**^
Tu	25.6 ± 3.7	0.91 ± 0.09	13.2 ± 4.4	25.6 ± 7.3	26.1 ± 3.7	0.89 ± 0.10	8.3 ± 2.5^**^	38.5 ± 7.2^**^
Salar	26.8 ± 4.0	0.93 ± 0.09	13.6 ± 5.0	26.9 ± 7.7	26.3 ± 4.3	0.88 ± 0.11^**^	8.0 ± 3.3^**^	39.2 ± 8.6^**^
Mongol	26.8 ± 4.3	0.92 ± 0.11	13.2 ± 4.9	24.8 ± 6.3	26.6 ± 4.7	0.86 ± 0.11^**^	7.8 ± 3.1^**^	37.1 ± 7.8^**^
Han	25.5 ± 3.5	0.89 ± 0.09	12.8 ± 4.5	24.0 ± 6.6	25.1 ± 3.4	0.84 ± 0.09^**^	7.3 ± 2.5^**^	36.1 ± 6.6^**^
Hui	25.7 ± 3.9	0.88 ± 0.10	12.4 ± 4.8	23.7 ± 7.8	25.6 ± 4.2	0.84 ± 0.10^**^	7.4 ± 2.9^**^	36.7 ± 7.9^**^
Kazakh	26.7 ± 3.4	0.92 ± 0.08	13.0 ± 3.5	24.6 ± 4.9	27.7 ± 4.8	0.88 ± 0.10^**^	8.0 ± 2.8^**^	39.9 ± 7.3^**^
Dongxiang	24.9 ± 3.4	0.86 ± 0.10	12.2 ± 4.4	21.8 ± 5.5	25.2 ± 3.8	0.83 ± 0.10^**^	7.0 ± 2.5^**^	34.5 ± 7.1^**^
Tibetan	26.5 ± 4.2	0.91 ± 0.10	12.5 ± 4.6	23.5 ± 6.1	25.8 ± 3.8	0.82 ± 0.09^**^	7.3 ± 2.6^**^	35.8 ± 6.6^**^
Bonan	24.6 ± 3.4	0.85 ± 0.09	11.5 ± 4.8	20.1 ± 5.9	25.1 ± 3.7	0.85 ± 0.09	7.1 ± 2.4^**^	34.9 ± 6.3^**^
*F* Value	11.58^△△^	20.09^△△^	7.73^△△^	18.67^△△^	14.97^△△^	13.70^△△^	11.91^△△^	19.86^△△^

Between-sex differences were assessed via the Mann-Whitney *U* test (** *P* < 0.01). *F* denotes the ANOVA statistic for comparisons across ethnic groups (△△ *P* < 0.01). BMI, kg/m²; WC, m; VFL, unitless (arbitrary units); BFP, %. Normally distributed continuous variables are presented as the means ± standard deviations; skewed continuous variables are presented as medians (interquartile range); and categorical variables are presented as *n* (%). The same conventions are used throughout unless otherwise specified

### Baseline characteristics of ethnic groups in Northwest China by obesity phenotype

**Fig. 1:**
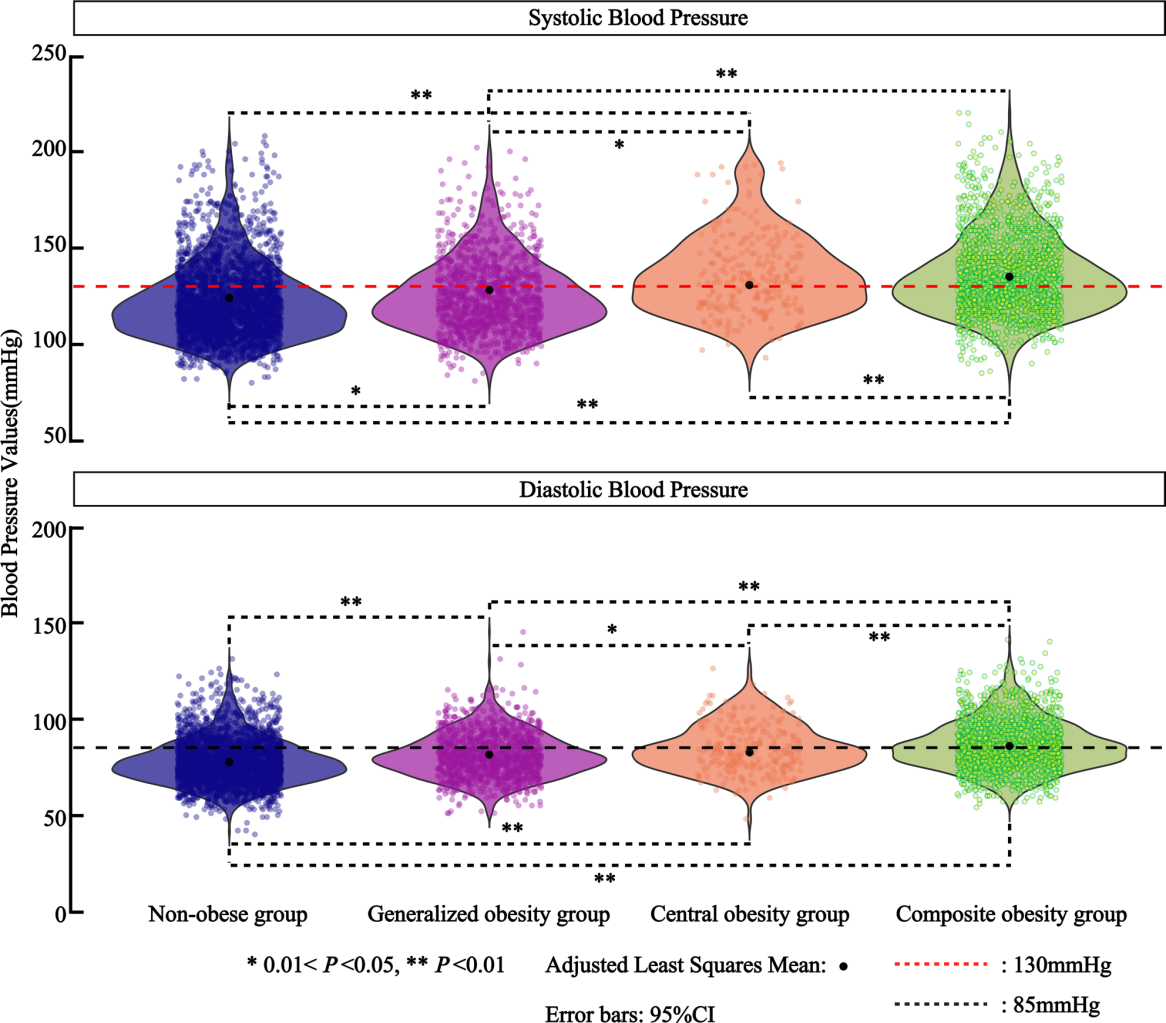
Adjusted mean blood pressure and distribution across obesity phenotypes on the basis of linear models

We further analysed the baseline characteristics of the participants by obesity phenotype (Table 2). Statistically significant differences were observed in age, sex, obesity-related indicators, ethnicity, and the prevalence of hypertension across the four obesity phenotypes (*P* < 0.01). Therefore, age, sex, and ethnicity were adjusted for as potential confounders in subsequent analyses. The hypertension prevalence rates were 65.5% (*n* = 1206) in the composite obesity group, 60.8% (*n* = 155) in the central obesity group, 43.2% (*n* = 523) in the generalized obesity group, and 35.5% (*n* = 1023) in the non-obese group. Analysis of the adjusted least-squares means revealed that both systolic and diastolic blood pressures were highest in the composite obesity group, followed by the central obesity, generalized obesity, and non-obesity groups(Fig. 1).

**Table 2:** Baseline characteristics of ethnic groups by obesity phenotype

	Composite Obesity Group	Central Obesity Group	Generalized Obesity Group	Non-Obese Group
Age^**^, n (%)				
18–44years	399(21.7)	32(12.5)	455(37.6)	1189(41.3)
45–59 years	843(45.8)	89(34.9)	583(48.1)	979(34.0)
≥ 60 years	598(32.5)	134(52.5)	173(14.3)	712(24.7)
Sex^**^, n (%)				
Male	1095(59.5)	242(94.9)	224(18.5)	1212(42.1)
Female	745(40.5)	13(5.1)	987(81.5)	1668(57.9)
Obesity-related indicators^**^				
BMI, kg/m^2^	29.9(28.1–31.7)	25.3(24.4–26.8)	26.9(26.0–28.0)	22.9(21.2–24.3)
WC, m	0.97(0.93–1.02)	0.93(0.91–0.95)	0.88(0.84–0.90)	0.80(0.75–0.84)
VFL	14.0(11.0–17.0)	14.0(12.0–16.0)	9.0(8.0–9.0)	7.0(5.0–9.0)
BFP, %	32.6(27.6–44.3)	22.8(21.4–24.6)	38.4(36.5–40.7)	26.5(19.7–33.0)
Prevalence^**^, n (%)				
Hypertension	1206(65.5)	155(60.8)	523(43.2)	1023(35.5)
Ethnicity^**^, n (%)				
Yugur	161(8.8)	7(2.7)	66(5.5)	128(4.4)
Tu	100(5.4)	25(9.8)	51(4.2)	128(4.4)
Salar	163(8.9)	33(12.9)	84(6.9)	125(4.3)
Mongol	360(19.6)	33(12.9)	188(15.5)	367(12.7)
Han	241(13.1)	50(19.6)	197(16.3)	518(18.0)
Hui	342(18.6)	38(14.9)	242(20.0)	601(20.9)
Kazakh	102(5.5)	8(3.1)	68(5.6)	85(3.0)
Dongxiang	162(8.8)	26(10.2)	157(13.0)	484(16.8)
Tibetan	131(7.1)	19(7.5)	77(6.4)	173(6.0)
Bonan	78(4.2)	16(6.3)	81(6.7)	271(9.4)

Group differences were compared across obesity phenotypes; ** *P* < 0.01

### Association between obesity phenotypes and hypertension

Table 3 presents the results of the Poisson regression analysis with robust variance, with the non-obese group used as the reference. Formal tests for interaction indicated significant effect modification by sex (*P* < 0.05), age (*P* < 0.01), and ethnicity (*P* < 0.01). Consequently, subsequent analyses were stratified by these factors. According to the fully adjusted model, the PR for hypertension decreased in the composite obesity and central obesity groups, whereas no reduction was observed in the generalized obesity group; the reference group had lower PRs than the other obesity groups did. In the model adjusted for only six variables, the composite obesity group presented higher PRs for hypertension than the other obesity groups did, except for the Yugur and Tu ethnic groups. Notably, in the 18–44 years age group, the PR for hypertension in the composite obesity group was substantially greater than that in the non-obese group.

**Table 3:** Association between obesity phenotypes and hypertension across different covariate adjustments

	Composite Obesity Group	Central Obesity Group	Generalized Obesity Group	Non-Obese Group
Unadjusted PR	1.85(1.74–1.96)^**^	1.71(1.53–1.91)^**^	1.22(1.12–1.32)^**^	Ref.
Fully adjusted PR	1.62(1.53–1.73)^**^	1.29(1.16–1.45)^**^	1.28(1.18–1.39)^**^	Ref.
^a^ Sex^△^				
Male	1.49(1.38–1.62)^**^	1.27(1.12–1.43)^**^	1.25(1.09–1.44)^**^	Ref.
Female	1.70(1.55–1.86)^**^	1.48(1.01–2.18)^*^	1.32(1.19–1.46)^**^	Ref.
^b^ Age^△△^				
18–44 years	2.09(1.79–2.44)^**^	1.40(0.90–2.18)	1.65(1.38–1.96)^**^	Ref.
45–59 years	1.63(1.48–1.79)^**^	1.55(1.29–1.85)^**^	1.19(1.05–1.34)^**^	Ref.
≥ 60 years	1.35(1.24–1.47)^**^	1.11(0.96–1.28)	1.18(1.04–1.34)^**^	Ref.
^c^ Ethnicity^△△^				
Yugur	1.46(1.19–1.79)^**^	1.58(1.15–2.18)^**^	0.96(0.68–1.34)	Ref.
Tu	1.22(0.99–1.48)	0.73(0.48–1.13)	1.23(0.95–1.59)	Ref.
Salar	1.87 (1.39–2.52)^**^	1.34(0.90–2.01)	1.71(1.24–2.37)^**^	Ref.
Mongol	1.87(1.56–2.25)^**^	1.37(0.97–1.93)	1.55(1.22–1.99)^**^	Ref.
Han	1.44(1.25–1.66)^**^	1.37(1.10–1.70)^**^	1.29(1.08–1.53)^**^	Ref.
Hui	1.68(1.48–1.90)^**^	1.55(1.23–1.94)^**^	1.27(1.07–1.50)^**^	Ref.
Kazakh	1.88(1.35–2.62)^**^	1.71(1.01–2.89)^*^	1.05(0.66–1.68)	Ref.
Dongxiang	1.78(1.48–2.13)^**^	0.90(0.54–1.48)	1.29(1.02–1.61)^*^	Ref.
Tibetan	1.37(1.05–1.79)^*^	1.02(0.61–1.68)	0.77(0.48–1.23)	Ref.
Bonan	1.88(1.46–2.43)^**^	1.84(1.16–2.92)^*^	1.52(1.13–2.05)^**^	Ref.

Data are presented as the prevalence ratios (PRs) and 95% confidence intervals (95%CIs). Significance of interaction effects: (Δ 0.01 < *P* < 0.05; ΔΔ *P* < 0.01). Significance of differences: * 0.01 < *P* < 0.05; ** *P* < 0.01. The fully adjusted model controls for sex, age, and ethnicity

^a^ adjusted for age and ethnicity

^b^ adjusted for sex and ethnicity; **c**, adjusted for sex and age

All adjusted models additionally control for educational attainment, physical activity level, smoking status, and alcohol consumption

### Association between obesity-related indicators and hypertension

The logistic regression analysis results (Table 4) revealed that all obesity-related indicators were positively associated with the odds of hypertension. After multivariable adjustment (Model 2), BMI, WC, VFL, and BFP were independently associated with hypertension. Analysis of continuous variables indicated that each 1 kg/m² increase in BMI was associated with a 13% increase in odds of hypertension (OR = 1.13; 95% CI: 1.11–1.15); each 0.01 m increase in WC was associated with a 6% increase in odds (OR = 1.06; 95% CI: 1.05–1.06); each 1-level increase in VFL was associated with a 14% increase in odds (OR = 1.14; 95% CI: 1.12–1.17); and each 1% increase in BFP was associated with a 7% increase in odds (OR = 1.07; 95% CI: 1.06–1.08). Analysis by quartile revealed that, compared with the lowest quartile (Q1), the highest quartile (Q4) was associated with significantly higher odds of hypertension: the BMI Q4 group had a 2.37-fold increase in the odds (OR = 3.37; 95% CI: 2.88–3.94); the WC Q4 group had a 3.38-fold increase (OR = 4.38; 95% CI: 3.71–5.17); the VFL Q4 group had a 3.16-fold increase (OR = 4.16; 95% CI: 3.40–5.09); and the BFP Q4 group had a 3.88-fold increase (OR = 4.88; 95% CI: 3.95–6.03). Notably, the odds ratio for the highest BFP quartile was the highest among all indicators, presenting a marked contrast to the ORs observed for BMI, WC, and VFL.

**Fig. 2:**
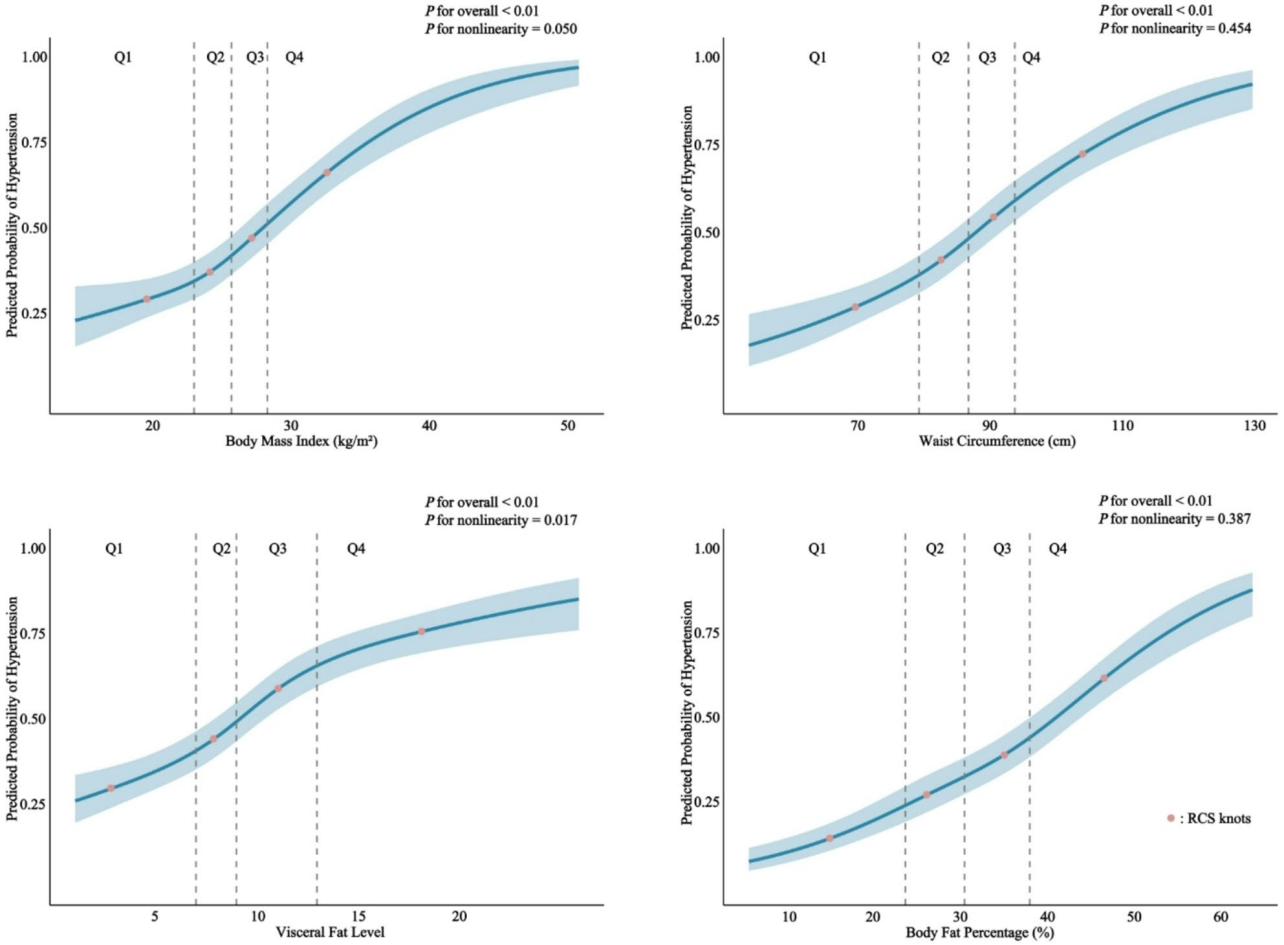
Dose‒response relationships between obesity-related indicators and hypertension among ethnic groups in Northwest China

The RCS analysis (Fig. 2) further revealed dose‒response relationships between obesity-related indicators and hypertension. Specifically, both WC and BFP demonstrated linear positive associations with hypertension (*P for nonlinearity* = 0.454\0.387). Notably, both VFL and BMI exhibited significant nonlinear associations with hypertension (*P for nonlinearity* = 0.017\0.050). The odds of hypertension increased gradually with rising BFP but rose more rapidly once BFP exceeded 35.3%(the third knot). FDR correction for multiple testing confirmed that the robust associations of WC, VFL, BFP, and BMI with hypertension were not due to chance, with all FDR-adjusted *P* values remaining statistically significant (Table 5).

**Table 4:** Results of logistic regression analysis of the associations between obesity-related indicators and hypertension

	Model 1	Model 2
BMI	1.15(1.13–1.16)	1.13(1.11–1.15)
Q1(≤ 23.1)	Ref.	Ref.
Q2(23.2–25.7)	1.57(1.36–1.82)	1.36(1.17–1.59)
Q3(25.8–28.3)	2.19(1.89–2.53)	1.85(1.59–2.16)
Q4(≥ 28.4)	4.01(3.45–4.65)	3.37(2.88–3.94)
* P* value	< 0.01	< 0.01
WC	1.07(1.07–1.08)	1.06(1.05–1.06)
Q1(≤ 79.5)	Ref.	Ref.
Q2(79.6–86.9)	2.08(1.79–2.43)	1.68(1.43–1.97)
Q3(87.0–93.9)	3.40(2.93–3.97)	2.56(2.19–3.00)
Q4(≥ 94)	6.35(5.43–7.43)	4.38(3.71–5.17)
* P* value	< 0.01	< 0.01
VFL	1.16(1.15–1.18)	1.14(1.12–1.17)
Q1(≤ 7.0)	Ref.	Ref.
Q2(7.1–8.9)	2.24(1.93–2.60)	1.75(1.50–2.05)
Q3(9–12.9)	3.20(2.78–3.69)	2.63(2.24–3.09)
Q4(≥ 13)	5.41(4.68–6.27)	4.16(3.40–5.09)
* P* value	< 0.01	< 0.01
BFP	1.03(1.02–1.03)	1.07(1.06–1.08)
Q1(≤ 23.9)	Ref.	Ref.
Q2(24.0–30.6)	1.54(1.34–1.78)	1.88(1.61–2.20)
Q3(30.7–38.1)	1.16(1.00–1.33)	2.59(2.13–3.15)
Q4(≥ 38.2)	2.02(1.75–2.34)	4.88(3.95–6.03)
* P* value	< 0.01	< 0.01

Data are presented as odds ratios (ORs) with 95% confidence intervals (95% CIs). Model 1: unadjusted; Model 2: adjusted for sex, age, ethnicity, educational attainment, physical activity level, smoking status, and alcohol consumption

**Table 5:** Multiple testing of obesity-related indicators with hypertension using false discovery rate correction

Variable	Univariate *P*-value	Univariate FDR-adjusted *P* value	Multivariate *P* value	Multivariate FDR-adjusted *P* value	VIF
WC	3.02 × 10^− 129^	1.21 × 10^− 128^	7.21 × 10^− 22^	1.44 × 10^− 21^	3.42
VFL	1.62 × 10^− 126^	3.25 × 10^− 126^	1.61 × 10^− 32^	6.46 × 10^− 32^	2.75
BFP	7.49 × 10^− 24^	7.49 × 10^− 24^	6.19 × 10^− 12^	8.25 × 10^− 12^	2.32
BMI	1.33 × 10^− 83^	1.78 × 10^− 83^	3.89 × 10^− 11^	3.89 × 10^− 11^	4.80

All FDR-adjusted *P* values were below the significance threshold of 0.01

## Discussion

This study revealed that composite obesity identifies individuals with hypertension more effectively than either central or generalized obesity alone and comprehensively assessed the potential associations between obesity-related indicators and hypertension. The results demonstrated statistically significant differences in obesity indicators among ethnic groups in Northwestern China. Males had a significantly greater VFL than females did, whereas their BFP was statistically significantly lower. Compared with that in the non-obese group, the hypertension incidence was 28% greater in the generalized obesity group, 29% greater in the central obesity group, and 62% greater in the composite obesity group. Consistent positive associations were observed between BMI, WC, VFL, and BFP and hypertension, and these associations remained persistent across different logistic regression models and RCS analyses, underscoring the robustness of obesity-related indicators as predictors of hypertension risk.

Compared with females, males had significantly greater VFL but lower BFP. This pattern suggests that sex-based differences in adiposity distribution are markedly amplified by the combined effects of high-fat diets, genetic background, and specific lifestyle factors prevalent in Northwestern China [29, 30]. It may be argued that men in Northwestern China who do not appear “obese” by conventional measures may still face substantially elevated risks of metabolic diseases, warranting heightened clinical attention. This study revealed significant ethnic heterogeneity. After adjusting for covariates, the Kazakh and Bonan ethnic groups showed a greater ability of composite obesity to identify hypertension than the other groups did, whereas among the Tu ethnic group, the difference in hypertension incidence between the composite obesity group and non-obese group was not statistically significant. This study adds to the growing consensus on the importance of ethnic heterogeneity, suggesting that tailored strategies for hypertension prevention and control could be more effective than uniform approaches. The thrifty genotype hypothesis proposes that energy-conserving adaptations increase susceptibility to visceral adiposity and insulin resistance in contemporary obesogenic environments, thereby contributing to ethnic heterogeneity [31]. Ethnic variations in sodium intake patterns and visceral to peripheral fat distributions further intensify metabolic disparities across populations [32]. Recent studies have also indicated variations in the obesity-hypertension relationship across racial and regional lines. For example, abdominal visceral fat and lean body mass have been shown to strongly discriminate hypertension risk in European and American populations [33, 34], whereas body roundness index, BMI, and WC are considered effective predictors in Asian populations [35, 36]. The present study further supports the important role of ethnic heterogeneity in the relationship between obesity and hypertension.

This study revealed a nonlinear association between VFL and hypertension risk. This pattern suggests a potential threshold effect of visceral fat accumulation, beyond which the risk of hypertension increases markedly. Specifically, the OR for VFL in the highest quartile (Q4) was 4.16 (95% CI, 3.40–5.09), supporting the concept of “normal-weight obesity” [37]—even in individuals with a normal BMI, elevated VFL or BFP are associated with a significantly increased cardiometabolic risk. Initially, adipose expansion is accommodated by angiogenesis and insulin-mediated lipid storage, with limited metabolic disturbance [38]. Beyond a critical visceral fat threshold, adipocyte hypertrophy induces relative hypoxia (HIF-1α activation), macrophage recruitment, and inflammatory cytokine/FFA spillover, impairing insulin signalling [39, 40]. These changes heighten sympathetic tone and renal sodium retention, accelerating hypertension and cardiometabolic disease [41]. In a prospective study by Zhao et al. [42], which included 10,265 participants without baseline hypertension in Luoyang, 2,027 new hypertension cases occurred during 6 years of follow-up. Individuals with a WC increase greater than 5% had a 34% greater risk of hypertension in men and a 28% greater risk in women. Another prospective study of 6096 participants without hypertension [43] revealed that the central obesity group had a 79% greater risk of developing hypertension than the normal group did (95% CI, 1.36–2.35). These findings corroborate the accuracy of the present cross-sectional analysis: in the unadjusted model, the hypertension incidence in the central obesity group was 1.71 times greater than that in the non-obese group (95% CI, 1.53–1.91); each 0.05–m increase in WC was associated with a 30% increase in hypertension risk.

This study confirmed positive associations of BMI and BFP with hypertension risk, although the relationship between body composition and metabolic syndrome is multidimensional. Li et al. [34] extracted data from 50,159 individuals in the National Health and Nutrition Examination Survey and revealed a potential association between lean body mass and hypertension: among participants with LBM below 43.21 kg, hypertension risk was negatively correlated with LBM. This finding, together with those of the present study, illustrates the complexity of body composition—both high fat mass and low LBM are independent risk factors for hypertension. These findings indicate that maintaining an appropriate LBM is as important as controlling obesity for the prevention of hypertension.

According to the guidelines recommended by the IDF, combining BMI and WC in obesity assessment helps uncover associations between different forms of obesity and various diseases [44]. Building on the IDF framework, this study incorporated two body composition indicators—BFP and VFL—combined with BMI and WC, respectively, to classify participants into four obesity phenotypes. This approach was explored to better characterize the association between obesity and hypertension. Yang et al. [14] reported that in an elderly Chinese population, other obesity phenotypes were associated with a lower risk of metabolic syndrome than the composite obesity group. Kim et al. [20] reported that among men in a Korean community, those with elevated WC and BMI values had a significantly greater probability of hypertension than did those in other groups. Our data, which demonstrated the strongest association between composite obesity and hypertension risk, further support these earlier conclusions.

The strengths of this study include the innovative combination of BMI with BFP and WC with VFL to establish a more comprehensive obesity phenotyping system that overcomes the limitations of traditional single indicators. This classification strategy allows more precise identification of associations between different obesity phenotypes and hypertension, particularly highlighting the high-risk nature of composite obesity. Furthermore, this is the first study to systematically demonstrate significant ethnic heterogeneity in obesity indicators in a multiethnic population in Northwestern China, suggesting that hypertension prevention and control require ethnic-specific strategies rather than uniform interventions. These insights provide an important foundation for precision public health.

Despite these valuable contributions, this study has several limitations. First, the cross-sectional design precludes causal inference between obesity and hypertension, and reverse causality cannot be ruled out. Second, although we adjusted for numerous potential confounders, residual confounding may remain due to unmeasured factors, such as detailed dietary sodium and potassium intake, sleep quality, and socioeconomic status. Third, although the overall sample size was large, some ethnic subgroups had relatively small sample sizes, which may affect the statistical power and stability of the association estimates. Finally, body composition was measured via bioelectrical impedance analysis, which is practical for large-scale epidemiological studies but is less accurate than more advanced imaging techniques such as Dual-energy X-ray Absorptiometry (DXA), or Magnetic Resonance Imaging (MRI).

## Conclusion

In summary, this study demonstrated that composite obesity is more effective than other obesity phenotypes in identifying individuals with hypertension and suggests that BMI, WC, VFL, and BFP may serve as potential indicators for hypertension screening. Furthermore, the significant ethnic variations observed highlight that ethnic heterogeneity should be considered in future prevention strategies. The validation of these findings and the development of tailored approaches require further investigation in diverse populations.

## Data Availability

The datasets generated and/or analyzed during the current study are available from the corresponding author on reasonable request.

## Abbreviations

95%CI: 95% confidence interval
ANOVA: Analysis of variance
BFP: Body fat percentage
BMI: Body mass index
DBP: Diastolic blood pressure
DXA: Dual-energy X-ray Absorptiometry
FDR: false discovery rate
IDF: International Diabetes Federation
MRI: Magnetic Resonance Imaging
OR: Odds ratio
RCS: Restricted cubic spline
PR: Prevalence ratio
SBP: Systolic blood pressure
VFL: Visceral fat level
VIF: Variance inflation factor
WC: Waist circumference
WHO: World Health Organization

## References

[1] Mills KT, Bundy JD, Kelly TN, Reed JE, Kearney PM, Reynolds K, et al. Global disparities of hypertension prevalence and control: a systematic analysis of population-based studies from 90 countries. Circulation. 2016;134(6):441–50. 27502908 10.1161/CIRCULATIONAHA.115.018912 PMC4979614

[2] Roth GA, Mensah GA, Johnson CO, Addolorato G, et al. Global burden of cardiovascular diseases and risk factors, 1990–2019: update from the GBD 2019 study. J Am Coll Cardiol. 2020;76(25):2982–3021. 33309175 10.1016/j.jacc.2020.11.010 PMC7755038

[3] WHO. Global report on hypertension: the race against a silent killer. Geneva: World Health Organization; 2023. pp. 5–8.

[4] Ma S, Yang L, Zhao M, Magnussen CG, Xi B. Trends in hypertension prevalence, awareness, treatment and control rates among Chinese adults, 1991–2015. J Hypertens. 2021;39(4):740–8. 33186320 10.1097/HJH.0000000000002698

[5] Nassar GM, Jameson R, Sathiyaraj S, Bidikian N, Villasmil Hernandez N, Sahay S. Recovery from kidney failure associated with chronic thromboembolic pulmonary hypertension following pulmonary thomboendarterectomy. Clin Kidney J. 2024;17(4):sfae047. 10.1093/ckj/sfae047. 38572501 10.1093/ckj/sfae047 PMC10986204

[6] Wang XF, Zhang XW, Liu YJ, Zheng XY, Su MR, Sun XH, et al. The causal effect of hypertension, intraocular pressure, and diabetic retinopathy: a Mendelian randomization study. Front Endocrinol (Lausanne). 2024;15:1304512. 38379860 10.3389/fendo.2024.1304512 PMC10877050

[7] Gui J, Li Y, Liu H, et al. Obesity-and lipid-related indices as a predictor of hypertension in Mid-aged and elderly chinese: A Cross-sectional study. BMC Geriatr. 2024;24(1):77. 38245677 10.1186/s12877-023-04650-2 PMC10800050

[8] Hu X, Han P, Liu Y. Metabolic status and hypertension: the impact of insulin resistance-related indices on blood pressure regulation and hypertension risk. J Am Nutr Assoc. 2025;44(6):487–97. 39791865 10.1080/27697061.2025.2450711

[9] Grillo MA, Mariani G, Ferraris JR. Prematurity and low birth weight in neonates as a risk factor for obesity, hypertension, and chronic kidney disease in pediatric and adult age. Front Med (Lausanne). 2022;8:769734. 35186967 10.3389/fmed.2021.769734 PMC8850406

[10] Miura SI, Matsuo Y, Seumatsu Y. Renin-angiotensin-aldosterone system and its relation to hypertension. Hypertens Res. 2025;48(8):2209–17. 40437114 10.1038/s41440-025-02229-5

[11] World Obesity Federation. World obesity atlas 2025. London: World Obesity Federation; 2025.

[12] Vecchié A, Dallegri F, Carbone F, Bonaventura A, Liberale L, Portincasa P, et al. Obesity phenotypes and their Paradoxical association with cardiovascular diseases. Eur J Intern Med. 2018;48:6–17. 29100895 10.1016/j.ejim.2017.10.020

[13] Blüher M. Obesity: global epidemiology and pathogenesis. Nat Rev Endocrinol. 2019;15(5):288–98. 30814686 10.1038/s41574-019-0176-8

[14] Yang M, Zhang Y, Zhao W, Ge M, Sun X, Zhang G, et al. Individual and combined associations of body mass index and waist circumference with components of metabolic syndrome among multiethnic middle-aged and older adults: A cross-sectional study. Front Endocrinol (Lausanne). 2023;14:1078331. 36909310 10.3389/fendo.2023.1078331 PMC9992890

[15] Li YL, Gong L, Sun SY, et al. Body composition of the central obese population in the Northwest ethnic corridor of China. Acta Anthropol Sin. 2025,44(04):651–60.

[16] Simati S, Kokkinos A, Dalamaga M, Argyrakopoulou G. Obesity paradox: fact or fiction? Curr Obes Rep. 2023;12(2):75–85. 36808566 10.1007/s13679-023-00497-1

[17] Correa-Rodríguez M, González-Ruíz K, Rincón-Pabón D, Izquierdo M, García-Hermoso A, Agostinis-Sobrinho C, et al. Normal-weight obesity is associated with increased cardiometabolic risk in young adults. Nutrients. 2020;12(4):1106. 32316150 10.3390/nu12041106 PMC7230158

[18] Sun JY, Su Z, Shen H, Hua Y, Sun W, Kong XQ. Abdominal fat accumulation increases the risk of high blood pressure: evidence of 47,037 participants from Chinese and US National population surveys. Nutr J. 2024;23(1):153. 39623430 10.1186/s12937-024-01058-5 PMC11610192

[19] Bouchi R, Takeuchi T, Akihisa M, Ohara N, Nakano Y, Nishitani R, et al. High visceral fat with low subcutaneous fat accumulation as a determinant of atherosclerosis in patients with type 2 diabetes. Cardiovasc Diabetol. 2015;14:136. 26445876 10.1186/s12933-015-0302-4 PMC4597374

[20] Kim H, Kim K, Shin S. Cardiometabolic risk factor in obese and normal weight individuals in community dwelling men. Int J Environ Res Public Health. 2020;17(23):8925. 33266289 10.3390/ijerph17238925 PMC7729436

[21] Chen L, Zhang J, Zhou N, Weng JY, Bao ZY, Wu LD. Association of different obesity patterns with hypertension in US male adults: a cross-sectional study. Sci Rep. 2023;13(1):10551. 37386040 10.1038/s41598-023-37302-x PMC10310720

[22] Xi HJ, Chen Z. Anthropometric methods. 2nd ed. Beijing: Science; 2010.

[23] Shao XQ. Anthropometry manual. Shanghai: Shanghai Lexicographical Publishing House; 1985. pp. 224–40.

[24] WHO.The Asia. -Pacific perspective: redefining obesity and its treatment. Sydney: Health Communications Australia; 2000.

[25] National Cholesterol Education Program Expert Panel on Detection. Evaluation and treatment of high blood cholesterol in Adults. Third report of the National cholesterol education program expert panel on detection, evaluation, and treatment of high bloodcholesterol in adults (Adult treatment panel lll) final report. Circulation. 2002;106:3143. 12485966

[26] Mare KU, Aychiluhm SB, Mulaw GF, et al. Non-adherence to antenatal iron supplementation and its determinants among pregnant women in 35 sub-saharan African countries: a generalized linear mixed-effects modeling with robust Poisson regression analysis. BMC Pregnancy Childbirth. 2024;24(1):872. 39732634 10.1186/s12884-024-07105-7 PMC11681747

[27] Jakobsen JC, Gluud C, Wetterslev J, Winkel P. When and how should multiple imputation be used for handling missing data in randomized clinical trials - a practical guide with flowcharts. BMC Med Res Methodol. 2017;17(1):162. 29207961 10.1186/s12874-017-0442-1 PMC5717805

[28] Kim JH. Multicollinearity and misleading statistical results. Korean J Anesthesiol. 2019;72(6):558–69. 31304696 10.4097/kja.19087 PMC6900425

[29] Blaak E. Gender differences in fat metabolism. Curr Opin Clin Nutr Metab Care. 2001;4(6):499–502. 11706283 10.1097/00075197-200111000-00006

[30] Vettori A, Pompucci G, Paolini B, Del Ciondolo I, Bressan S, Dundar M, et al. Genetic background, nutrition and obesity: a review. Eur Rev Med Pharmacol Sci. 2019;23(4):1751–61. 30840300 10.26355/eurrev_201902_17137

[31] Wells JCK. Natural selection and human adiposity: crafty genotype, thrifty phenotype. Philos Trans R Soc Lond B Biol Sci. 2023;378(1885):20220224. 37482776 10.1098/rstb.2022.0224 PMC10363707

[32] Ramessur V, Hunma S, Joonas N, Ramessur BN, Schutz Y, Montani JP, et al. Visceral-to-peripheral adiposity ratio: a critical determinant of sex and ethnic differences in cardiovascular risks among Asian Indians and African Creoles in Mauritius. Int J Obes (Lond). 2024;48(8):1092–102. 38615158 10.1038/s41366-024-01517-3 PMC11281908

[33] Liu J, Fox CS, Hickson D, Bidulescu A, Carr JJ, Taylor HA. Fatty liver, abdominal visceral fat, and cardiometabolic risk factors: the Jackson heart study. Arterioscler Thromb Vasc Biol. 2011;31(11):2715–22. 21885852 10.1161/ATVBAHA.111.234062 PMC3228266

[34] Li S, Jiao H, Yang J, Li Y, Zhang J, Liu X, Xue Y. Association between lean body mass and hypertension: A cross-sectional study of 50 159 NHANES participants. J Clin Hypertens (Greenwich). 2023;25(10):957–64. 37614028 10.1111/jch.14710 PMC10560971

[35] Zhan Q, An Q, Zhang F, Zhang T, Liu T, Wang Y. Body roundness index and the risk of hypertension: a prospective cohort study in Southwest China. BMC Public Health. 2024;24(1):2539. 39294669 10.1186/s12889-024-20049-z PMC11411781

[36] Wang Q, Song X, Du S, Du W, Su C, Zhang J, et al. Multiple trajectories of body mass index and waist circumference and their associations with hypertension and blood pressure in Chinese adults from 1991 to 2018: a prospective study. Nutrients. 2023;15(3):751. 36771457 10.3390/nu15030751 PMC9919034

[37] Ren H, Guo Y, Wang D, Kang X, Yuan G. Association of normal-weight central obesity with hypertension: a cross-sectional study from the China health and nutrition survey. BMC Cardiovasc Disord. 2023;23(1):120. 36890477 10.1186/s12872-023-03126-w PMC9996911

[38] Morigny P, Boucher J, Arner P, Langin D. Lipid and glucose metabolism in white adipocytes: pathways, dysfunction and therapeutics. Nat Rev Endocrinol. 2021;17(5):276–95. 33627836 10.1038/s41574-021-00471-8

[39] Jacks RD, Lumeng CN. Macrophage and T-cell networks in adipose tissue. Nat Rev Endocrinol. 2024;20(1):50–61. 37872302 10.1038/s41574-023-00908-2

[40] Huynh PM, Wang F, An YA. Hypoxia signalling in the adipose tissue. J Mol Cell Biol. 2025;16(8):mjae039. 39363240 10.1093/jmcb/mjae039 PMC11892559

[41] Hall JE, et al. Obesity, kidney dysfunction and hypertension: mechanistic links. Nat Rev Nephrol. 2019;15(6):367–85. 31015582 10.1038/s41581-019-0145-4 PMC7278043

[42] Zhao Y, Zhang M, Luo X, Wang C, Li L, Zhang L, et al. Association of 6-year waist circumference gain and incident hypertension. Heart. 2017;103(17):1347–52. 28389523 10.1136/heartjnl-2016-310760

[43] Niu J, Seo DC. Central obesity and hypertension in Chinese adults: a 12-year longitudinal examination. Prev Med. 2014;62:113–8. 24552844 10.1016/j.ypmed.2014.02.012

[44] Quah YV Jr, Poh BK, Ismail MN. Metabolic syndrome based on IDF criteria in a sample of normal weight and obese school children. Malays J Nutr. 2010;16(2):207–17. 22691926

